# Prognostic role of programmed death-ligand 1 expression in patients with biliary tract cancer: a meta-analysis

**DOI:** 10.18632/aging.102588

**Published:** 2019-12-27

**Authors:** Changjiang Lei, Xiulan Peng, Xiaojun Gong, Ying Fan, Shenglin Wu, Ning Liu, Lei Li, Jianbin Huang, Gang Zheng, Zhixiong Long

**Affiliations:** 1 Department of General Surgery, The Second Affiliated Hospital of Jianghan University, Wuhan, China; 2 Department of Oncology, The Second Affiliated Hospital of Jianghan University, Wuhan, Hubei 430000, China; 3 Department of Cardiology, The Second Affiliated Hospital of Jianghan University, Wuhan, Wuhan, Hubei 430000, China; 4 Department of Pharmacology, The Second Affiliated Hospital of Jianghan University, Wuhan, Wuhan, Hubei 430000, China

**Keywords:** meta-analysis, PD-L1, biliary tract cancer, survival

## Abstract

Previous studies investigated the prognostic role of programmed death-ligand 1 (PD-L1) expression in patients with biliary tract cancer (BTC); however, the results remained controversial. Therefore, we conducted the current meta-analysis with the aim of clarifying the association between PD-L1 expression and prognosis as well as with several important clinicopathological features of BTC. We searched PubMed, Embase, and Web of Science for relevant studies. Studies that detected PD-L1 expression in tumor cells by using immunohistochemistry (IHC) were selected. Pooled hazard ratios (HRs) and pooled odds ratios (ORs) with 95% confidence intervals (CIs) were calculated to estimate the correlations. In total, 15 independent studies with 1,776 patients were included in this meta-analysis. The pooled data demonstrated that high PD-L1 expression was associated with poor overall survival (n=15, HR=1.79, 95% CI=1.55–2.07, p<0.001). The correlation between PD-L1 expression and disease-free survival was not significant (n=6, HR=1.38, 95% CI=1.00–1.91, p=0.051). In addition, no significant correlation was observed between PD-L1 expression and clinical features in patients with BTC. Our study results showed that PD-L1 expression could play a pivotal role as an effective factor of poor prognosis in patients with BTC.

## INTRODUCTION

Biliary tract cancer (BTC) is the second most common cancer among hepatobiliary cancers, and it is a heterogeneous group of gastrointestinal tumors [1]. BTC includes gallbladder cancer (GBC), intrahepatic cholangiocarcinoma (iCCA), and extrahepatic cholangiocarcinoma (eCCA) [2]. GBC is the most common BTC and is aggressive. Tumor stage is the strongest prognostic factor for patients with GBC [3]. Cholangiocarcinomas (CCAs) are tumors originating from the epithelium of the bile duct and are further classified as iCCA and eCCA [4]. GBC, iCCA, and eCCA are distinct entities because of their different tumor biology and treatment guidelines [5]. The latest National Comprehensive Cancer Network (NCCN) guidelines for hepatobiliary cancers provide different staging and treatment strategies for GBC, iCCA, and eCCA. Overall, the prognosis of BTC is dismal because 60–70% of cases are diagnosed at the advanced stage of disease [6]. The median overall survival (OS) of patients with advanced BTC does not exceed 12 months [7]. This poor prognosis could be partially attributed to a lack of efficient prognostic markers. Therefore, the identification of new and effective biomarkers that are correlated with BTC prognosis is very important and urgent.

Further understanding of the tumor immune microenvironment has led to the development of immunotherapy, which has garnered much attention in recent years [8, 9]. Programmed death-ligand 1 (PD-L1) is the main ligand of PD-1, and the interaction between PD-L1 and PD-1 is a major inhibitory pathway of immunosuppression in the tumor microenvironment [10]. In addition, previous studies explored the prognostic significance of PD-L1 in patients with BTC, with conflicting results [11–25]. For example, the results of some studies showed that PD-L1 overexpression in tumor cells predicted poor survival of patients with BTC [13, 21], whereas the results of other studies showed that PD-L1 was not a significant prognostic factor for BTC [14]. However, some researchers reported that high expression of PD-L1 was associated with superior survival outcomes [25]. Therefore, we comprehensively retrieved the relevant studies and conducted this meta-analysis to evaluate the association between PD-L1 expression and the prognosis and clinicopathological factors of patients with BTC. As BTC is a group of heterogeneous diseases, we also conducted subgroup analyses according to the different tumor types.

## RESULTS

### Search results

**Figure 1:**
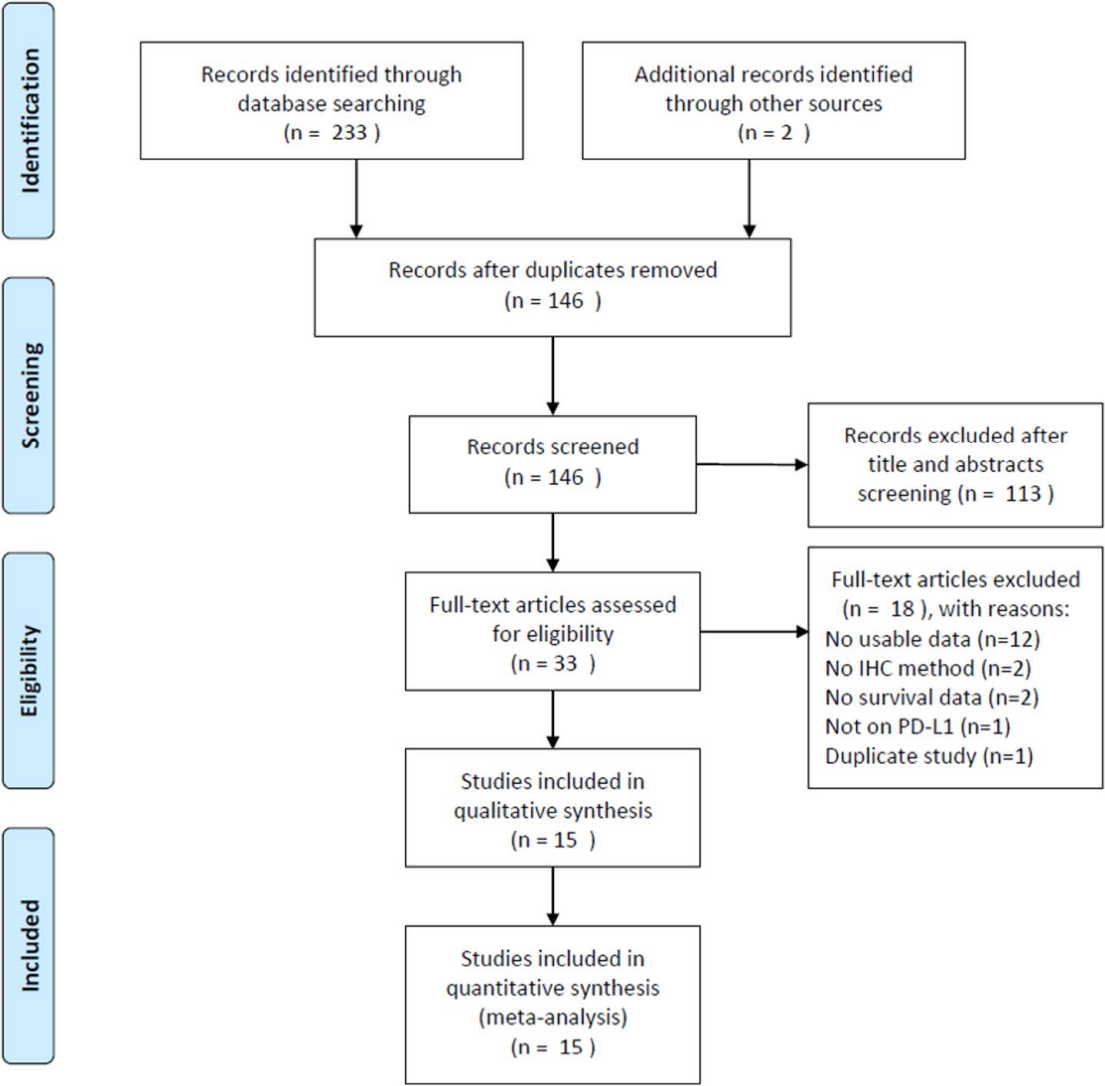
**Flow chart of literature search and study selection.**

The process of literature selection is presented in Figure 1. The initial literature search revealed 235 records, and after removing the duplicate papers, 146 articles were subjected to further screening. After examining the title and abstract, 113 reports were discarded. Subsequently, the full text of the remaining 33 studies were evaluated, and 18 studies were excluded owing to the following reasons: 12 studies did not provide usable data, 2 studies did not employ the IHC method, 2 studies did not provide any survival information, 1 study did not focus on PD-L1, and 1 study was overlapped. Finally, a total of 15 studies [11–25] comprising 1,776 patients were included for the meta-analysis.

### Characteristics of included studies

The characteristics of the included studies are shown in Table 1. The included studies were published between 2014 and 2019. All the studies used IHC to detect PD-L1 expression in tumor cells. The patients were enrolled from 6 different countries: China, Japan, Germany, the USA, Thailand, and Korea. Regarding the tumor type, 7 studies included patients with eCCA [11, 13, 15, 16, 18, 20, 25], 4 studies focused on patients with iCCA [12, 19, 21, 24], 3 studies reported on patients with iCCA and eCCA [14, 22, 23], and 1 study included patients with GBC [17]. Regarding the association between PD-L1 expression and prognosis, all 15 studies provided data about the association between PD-L1 and OS [11–25], and 6 studies also provided data on the association between PD-L1 expression and DFS [16, 17, 19–21, 25]. The NOS scores of the included 15 studies ranged from 6 to 8, indicating high-quality studies.

**Table 1:** Baseline characteristics of eligible studies for this meta-analysis.

**Study**	**Year**	**Patients, n**	**Country**	**Tumor type**	**Period**	**Age (years) Median/mean (range)**	**Ethnicity**	**Specimen**	**Detection method**	**Treatment**	**Cut-off value**	**Endpoint**	**NOS score**
Tamai	2014	91	Japan	eCCA	2000-2008	NA	Asian	Tissue	IHC	Surgical resection	Moderate or intense staining	OS	6
Gani	2016	54	USA	iCCA	1991-2011	Mean: 64	Caucasian	Tissue	IHC	Surgical resection	>5% tumor cells	OS	8
Ma	2017	70	China	eCCA	2009-2013	Mean: 62.5 Range: 33-83	Asian	Tissue	IHC	Surgical resection	>50% tumor cells	OS	8
Sangkhamanon	2017	46	Thailand	iCCA, eCCA	NA	Median: 57.5 Range: 45-76	Asian	Tissue	IHC	Surgical resection	>1% tumor cells	OS	6
Walter	2017	69	Germany	eCCA	2007-2015	NA	Caucasian	Tissue	IHC	Surgical resection	Score 3	OS	6
Kim	2018	34	USA	eCCA	1990-2015	Median: 67 Range: 42-86	Caucasian	Tissue	IHC	Surgical resection	>1% tumor cells	OS, DFS	8
Lin	2018	66	China	GBC	2009-2014	Median: 65 Range: 29-81	Asian	Tissue	IHC	Surgical resection	>5% tumor cells	OS, DFS	8
Ueno	2018	117	Japan	eCCA	1995-2006	Median: 71 Range: 44-87	Asian	Tissue	IHC	Surgical resection	Score 2	OS	8
Zhu	2018	192	China	iCCA	NA	NA	Asian	Tissue	IHC	Surgical resection	>5% tumor cells	OS, DFS	7
Ahn	2019	183	Korea	eCCA	2003-2013	Median: 68 Range: 41-83	Asian	Tissue	IHC	Surgical resection	>1% tumor cells	OS, DFS	8
Dong	2019	125	China	iCCA	2012-2013	Mean: 49 Range: 29-65	Asian	Tissue	IHC	Surgical resection	>5% tumor cells	OS, DFS	7
Kitano	2019	177	Japan	iCCA, eCCA	2005-2014	NA	Asian	Tissue	IHC	Surgical resection	>25% tumor cells	OS	7
Kriegsmann	2019	170	Germany	iCCA, eCCA	1995-2010	Median: 63 Range: 31-91	Caucasian	Tissue	IHC	Surgical resection	>1% tumor cells	OS	8
Lu	2019	320	China	iCCA	2005-2011	Median: 58	Asian	Tissue	IHC	Surgical resection	>5% tumor cells	OS	8
Yu	2019	62	China	eCCA	2015-2017	Mean: 60.8 Range: 22-81	Asian	Tissue	IHC	Surgical resection	Score 3	OS, DFS	8

NA, not available; iCCA, intrahepatic cholangiocarcinoma; eCCA, extrahepatic cholangiocarcinoma; GBC, gallbladder cancer; IHC, immunohistochemistry; OS, overall survival; DFS, disease-free survival; NOS, Newcastle-Ottawa scale.

### Correlation of PD-L1 expression with OS

**Figure 2:**
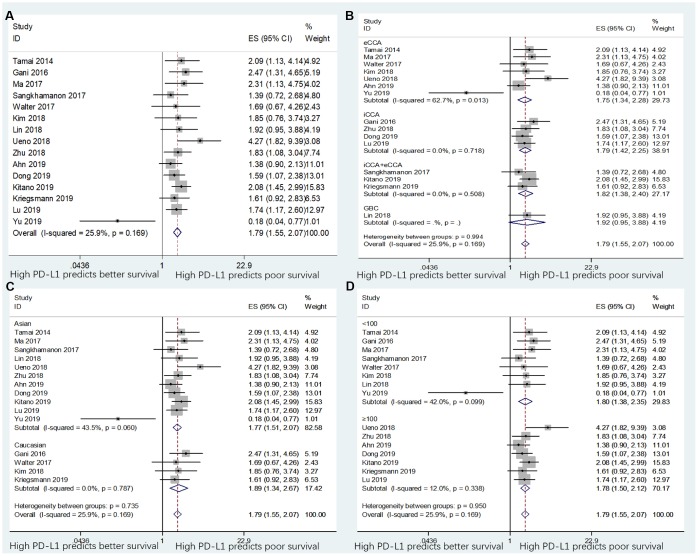
Forest plots for the association between PD-L1 expression and overall survival (OS) categorized by different subgroups: (**A**) the entire patient group; (**B**) patients with eCCA, iCCA, iCCA+eCCA, or GBC; (**C**) patients with Asian ethnicity or Caucasian ethnicity; and (**D**) studies with sample size ≥100 or sample size <100. Note: The right-side means “High PD-L1 predicts poor survival” and the left-side means “High PD-L1 predicts better survival”.

The data between PD-L1 expression and OS were extracted from all the 15 included studies with 1,776 patients [11–25]. The heterogeneity was not significant (*I*^2^=25.9%, p=0.167); therefore, a fixed-effects model was adopted. As shown in Table 2 and Figure 2A, the pooled data demonstrated that high PD-L1 expression was associated with poor OS (n=15, HR=1.79, 95% CI=1.55–2.07, p<0.001). In addition, subgroup analysis was performed for further investigation. On stratification by tumor type, ethnicity, and sample size, the data showed that PD-L1 remained a significant factor of poor OS in patients with eCCA (n=7, HR=1.73, 95% CI=1.08–2.75, p=0.022) and for patients with iCCA (n=4, HR=1.79, 95% CI=1.42–2.25, p<0.001), but not for patients with GBC (n=1, HR=1.92, 95% CI=0.95–3.88, p=0.069; Table 2 and Figure 2B). Moreover, PD-L1 expression was also a significant prognostic factor of poor OS irrespective of ethnicity (Table 2 and Figure 2C) and sample size (Table 2 and Figure 2D). Elevated PD-L1 expression was also a significant prognostic factor for OS in patients with BTC with different cut-off values of PD-L1 (Table 2 and Supplementary Figure 1A).

**Table 2:** Subgroup analyses of OS and DFS based on different factors.

**Survival outcome**	**Subgroup**	**Studies, n**	**Effects model**	**HR (95%CI)**	**p value**	**Heterogeneity**		**Meta-regression, p value**
						***I*^2^(%)**	**p value**	
OS	Total	15	Fixed	1.79 (1.55-2.07)	<0.001	25.9	0.167	
	Tumor type							0.820
	eCCA	7	Random	1.73 (1.08-2.75)	0.022	62.7	0.013	
	iCCA	4	Fixed	1.79 (1.42-2.25)	<0.001	0	0.718	
	iCCA+eCCA	3	Fixed	1.82 (1.38-2.40)	<0.001	0	0.508	
	GBC	1	-	1.92 (0.95-3.88)	0.069	-	-	
	Ethnicity							0.783
	Asian	11	Fixed	1.77 (1.51-2.07)	<0.001	43.5	0.06	
	Caucasian	4	Fixed	1.89 (1.34-2.67)	<0.001	0	0.787	
	Sample size							0.960
	<100	8	Fixed	1.80 (1.38-2.35)	<0.001	42	0.099	
	≥100	7	Fixed	1.78 (1.50-2.12)	<0.001	12	0.338	
	Cut-off value							0.166
	Cut-off value 5%	5	Fixed	1.80 (1.45-2.24)	<0.001	0	0.847	
	Cut-off value 1%	4	Fixed	1.49 (1.12-1.98)	0.001	0	0.917	
	Other cut-off values	6	Random	1.88 (1.14-3.10)	0.014	65.0	0.014	
DFS	Total	6	Random	1.38 (1.00-1.91)	0.051	64	0.016	
	Tumor type							0.271
	eCCA	3	Random	0.97 (0.53-1.78)	0.930	76	0.015	
	GBC	1	-	1.87 (0.95-3.68)	0.070	-	-	
	iCCA	2	Fixed	1.76 (1.28-2.42)	<0.001	0	0.752	
	Ethnicity							0.365
	Asian	5	Random	1.42 (0.93-2.18)	0.108	63.6	0.027	
	Caucasian	1	-	1.18 (0.95-1.47)	0.139	-	-	
	Sample size							0.288
	<100	3	Random	0.93 (0.40-2.16)	0.869	78.8	0.009	
	≥100	3	Fixed	1.64 (1.27-2.13)	<0.001	0	0.724	
	Cut-off value							0.425
	Cut-off value 5%	3	Fixed	1.78 (1.33-2.37)	<0.001	0	0.939	
	Cut-off value 1%	2	Fixed	1.22 (1.00-1.49)	0.045	0	0.458	
	Other cut-off values	1	-	0.16 (0.04-0.65)	0.011	-	-	

iCCA, intrahepatic cholangiocarcinoma; eCCA, extrahepatic cholangiocarcinoma; GBC, gallbladder cancer; OS, overall survival; DFS, disease-free survival.

### Correlation between PD-L1 expression and DFS

**Figure 3:**
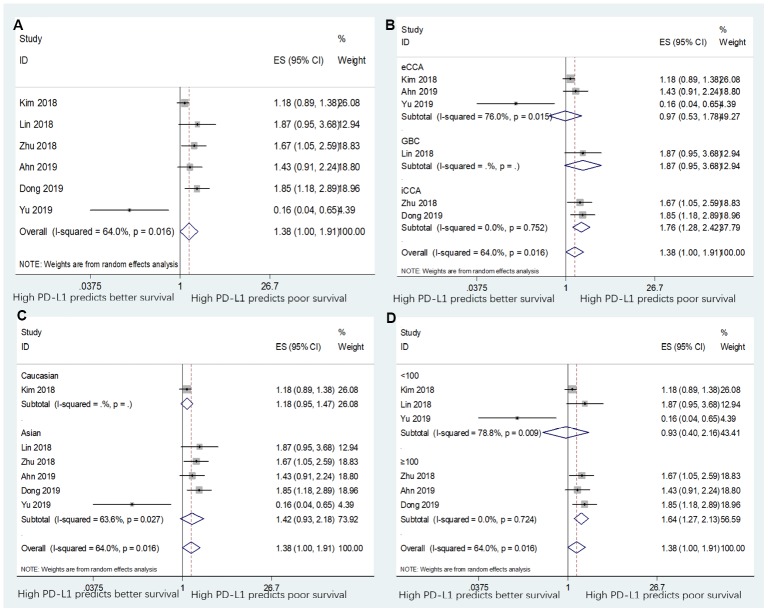
Forest plots for the association between PD-L1 expression and disease-free survival (DFS) categorized by different subgroups: (**A**) the entire patient group; (**B**) patients with eCCA, GBC, or iCCA; (**C**) patients with Asian ethnicity or Caucasian ethnicity; and (**D**) studies with sample size ≥100 or sample size <100. Note: The right-side means “High PD-L1 predicts poor survival” and the left-side means “High PD-L1 predicts better survival”.

Six studies with 662 patients provided the HRs for DFS [16, 17, 19–21, 25]. Because of significant heterogeneity (*I*^2^=65%, p=0.016), a random-effects model was applied. The pooled HR indicated that the correlation between PD-L1 expression and DFS was not significant (n=6, HR=1.38, 95% CI=1.00–1.91, p=0.051; Table 2 and Figure 3A). Subgroup analysis revealed that PD-L1 overexpression was associated with worse DFS in patients with iCCA (n=2, HR=1.76, 95% CI=1.28–2.42, p<0.001) and in studies with sample size ≥100 (n=3, HR=1.64, 95% CI=1.27–2.13, p<0.001; Table 2 and Figure 3B). However, high PD-L1 expression was not predictive of poor DFS in Asian and Caucasian patients (Table 2 and Figure 3C), in patients with eCCA and GBC (Table 2 and Figure 3B), and in studies with sample size <100 (Table 2 and Figure 3D). The pooled data also indicated that PD-L1 overexpression remained a significant prognostic factor for DFS using various cut-off values of PD-L1 (Table 2 and Supplementary Figure 1B).

### Association between PD-L1 expression and clinicopathological features

As GBC, iCCA, and eCCA are heterogeneous diseases and are considered different entities, we analyzed the correlation between PD-L1 expression and clinicopathological factors in the following 3 categories: iCCA, eCCA, and iCCA+eCCA. GBC was not analyzed because only 1 study was eligible. As shown in Table 3, the pooled ORs and 95% CIs showed that, for eCCA, there was no significant correlation between PD-L1 expression and sex (p=0.710), T stage (p=0.492), N stage (p=0.070), or tumor grade (p=0.126). In addition, for patients with iCCA, there was no significant association between PD-L1 expression and sex (p=0.651), tumor size (p=0.661), N stage (p=0.852), vascular invasion (p=0.116), or perineural invasion (p=0.529). For patients with eCCA and/or iCCA, there was no significant correlation between PD-L1 expression and sex (p=0.290), T stage (p=0.741), or N stage (p=0.174; Table 3).

**Table 3:** Meta-analysis of PD-L1 expression and clinicopathological features in BTC patients.

**Tumor type**	**Clinicopathological feature**	**Studies, n**	**Effects model**	**OR (95%CI)**	**P value**	**Heterogeneity**	
						***I*^2^(%)**	**p value**
eCCA	Sex (male vs female)	6	Fixed	1.09(0.69-1.72)	0.710	0	0.826
	T stage (T3-T4 vs T1-T2)	4	Fixed	0.80(0.43-1.50)	0.492	0	0.515
	N stage (N1 vs N0)	6	Fixed	1.48(0.97-2.27)	0.070	42.3	0.123
	Grading (G3 vs G1+G2)	3	Fixed	1.85(0.84-4.07)	0.126	22.2	0.277
iCCA	Sex (male vs female)	4	Fixed	1.11(0.71-1.73)	0.651	0	0.931
	Tumor size (≥5cm vs <5cm)	2	Random	0.73(0.18-2.98)	0.661	80.3	0.024
	N stage (N1 vs N0)	4	Random	1.22(0.15-9.91)	0.852	92.8	<0.001
	Vascular invasion (yes vs no)	4	Random	3.24(0.75-14.00)	0.116	82.4	0.001
	Perineural invasion (yes vs no)	3	Fixed	0.77(0.36-1.72)	0.529	0	0.706
iCCA+eCCA	Sex (male vs female)	2	Fixed	1.36(0.77-2.42)	0.290	9.3	0.294
	T stage (T3-T4 vs T1-T2)	2	Fixed	1.10(0.63-1.91)	0.741	41.9	0.190
	N stage (N1 vs N0)	3	Fixed	1.46(0.85-2.52)	0.174	0	0.586

### Sensitivity analysis and meta-regression analysis

**Figure 4:**
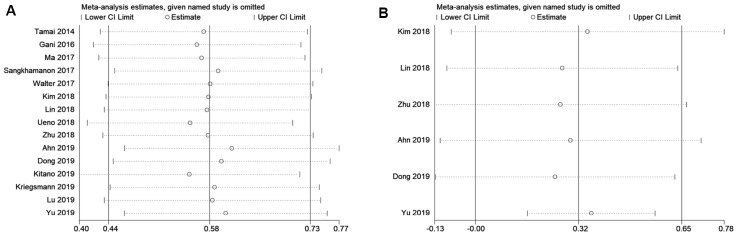
**Sensitivity analysis for the association between PD-L1 expression levels with BTC.**

Sensitivity analysis was performed to evaluate the stability of pooled HRs for OS and DFS. As shown in Figure 4, the results of the sensitivity analysis demonstrated high credibility of the pooled HRs. Meta-regression analysis showed that tumor type (p=0.820), ethnicity (p=0.783), sample size (p=0.960), and cut-off value (p=0.166) did not significantly contribute to heterogeneity of OS (Table 2). Similarly, meta-regression analysis also indicated that tumor type (p=0.271), ethnicity (p=0.365), sample size (p=0.288), and cut-off value (p=0.425) did not significantly contribute to heterogeneity of DFS (Table 2).

### Publication bias

**Figure 5:**
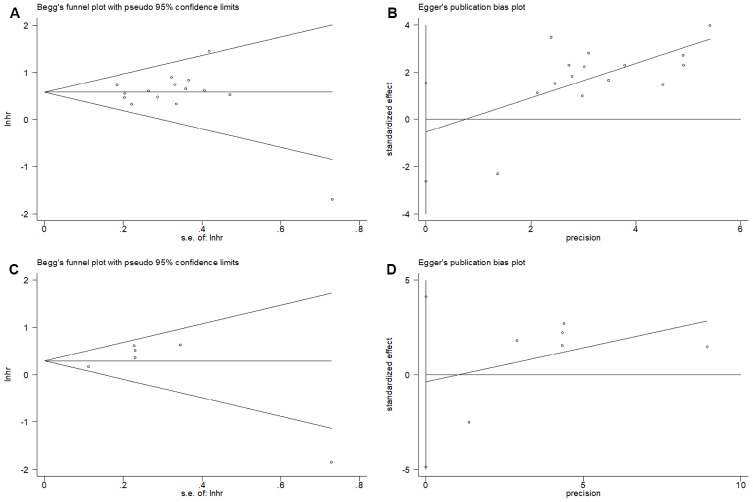
**Publication bias examination.** (**A**) Begg’s funnel plots assessing the publication bias for OS (p=0.921); (**B**) Egger’s test assessing the publication bias for OS (p=0.581); (**C**) Begg’s funnel plots assessing the publication bias for DFS (p=0.452); and (**D**) Egger’s test assessing the publication bias for DFS (p=0. 0.826).

The Begg’s funnel plots and Egger’s test were used to estimate the potential publication bias. The results showed that there was no significant publication bias for OS on the Begg’s test (p=0.921, Figure 5A) and Egger’s test (p=0.581, Figure 5B). Similarly, the Begg’s test (p=0.452, Figure 5C) and Egger’s test (p=0.826, Figure 5D) indicated no significant publication bias for DFS.

## DISCUSSION

BTCs are a diverse group of tumors and have poor prognosis owing to the advanced stage at the time of initial diagnosis [5]. BTC is associated with immune-related risk factors, and PD-L1 was an important mediating factor in the tumor immune microenvironment. In the present meta-analysis, survival data from 15 studies with 1,776 patients were integrated. The results demonstrated that PD-L1 overexpression was associated with poor OS but not poor DFS in patients with BTC. There was no significant correlation between PD-L1 expression and clinicopathological features in patients with either iCCA or eCCA. Sensitivity analysis, meta-regression analysis and publication bias tests suggested that the results were stable and credible. According to the 2019 NCCN guideline of hepatobiliary cancers, GBC, iCCA and eCCA are considered 3 different clinical entities, with different TNM staging and prognosis. BTCs are a heterogenous group of tumors including GBC, iCCA, and eCCA. Therefore, to conform to clinical application, we included patients with BTC, and performed subgroup analyses on GBC, iCCA, and eCCA, separately. This meta-analysis provides important implications for the prognosis of BTC and for all professional practitioners who are referring to NCCN guidelines.

PD-L1 is upregulated by many inflammatory mediators and cytokines within the tumor microenvironment [26]. The binding of PD-1/PD-L1 can inhibit T-cell activation, induce activated T-cell apoptosis, and negatively mediate the immune response [27]. Many clinical trials have evaluated the use of PD-1/PD-L1 inhibitors for treating gastrointestinal malignancies [28]. For patients with advanced-stage BTC, treatment options are limited [1]. Current evidence showed that adjuvant chemotherapy was associated with an improvement in OS of patients with BTC [29]. More recent studies showed that nivolumab had a manageable safety profile and signs of clinical activity in patients with unresectable or recurrent BTC [30]. Another recent study also showed that the adverse effects after nivolumab for metastatic BTC were controllable [31]. All these results imply that immune checkpoint inhibitors show promising clinical efficacy for patients with unresectable BTC.

The prognostic effect of PD-L1 expression has also been investigated in other types of cancers in a meta-analysis. A recent meta-analysis—based on data from 11 studies involving 1,697 cases—showed that PD-L1 overexpression could predict worse survival outcomes in patients with bladder cancer [32]. Another study [33] also showed that high expression of PD-L1 was associated with inferior OS in patients with colorectal cancer. In addition, elevated PD-L1 expression was positively correlated with lymph node metastasis [33]. A comprehensive meta-analysis of 50 studies with 11,383 patients demonstrated that PD-L1 expression on IHC was associated with poor OS and with several clinicopathological factors in patients with lung cancer [34]. In the present meta-analysis, the pooled data showed that high PD-L1 expression was predictive of poor OS, in line with the results of previous studies on other cancers. However, we did not identify any significant correlation between PD-L1 expression and any clinical factors in patients with BTC, which may be owing to the limited sample size while analyzing eCCA and iCCA.

Notably, since included studies used different cut-off values of tumor stage and age to divide patients for the analysis of correlation with PD-L1 expression, we did not perform meta-analysis on those two factors (tumor stage and age). Therefore, we examined each included study separately. A total of 11 included studies [11, 13, 15, 18–25] reported the correlation of PD-L1 expression and age of patients, using different cut-off values: 4 studies used 60 years [13, 20, 21, 25], 3 studies applied 65 years [11, 15, 19], one study selected 58 years [24], 63 years [23], 71 years [18], and mean±Standard Deviation (SD) [22], respectively. Except for one study [22] showed that the age of patients with different expression levels of PD-L1 in cancer cells was significantly different (p=0.03), other 10 studies [11, 13, 15, 18–21, 23–25] reported non-significant association between PD-L1 expression and age of patients. Those results suggested the connection between PD-L1 expression and age may not be significant in patients with BTC. It is noteworthy that the correlation of PD-L1 and aging have been explored in relevant recent studies. A recent study from US Food and Drug Administration (FDA) showed that patient-reported outcomes in patients with advanced lung cancer receiving anti-PD-1/PD-L1 therapy were not significantly different between younger and older patients (cut-offed by 70 years) [35]. Moreover, a meta-analysis including 27 randomized controlled trials (RCTs) with 17,546 patients demonstrated that immune checkpoint inhibitors (ICIs) could not significantly improve OS and PFS compared with controls in cancer patients aged over 75 years [36].

Although we strictly performed this meta-analysis according to the PRISMA guidelines and selected eligible studies with uniform criteria, the study has several limitations. First, although all included studies used IHC to detect PD-L1 expression, the cut-off values defining low/high PD-L1 levels were different, which may result in heterogeneity. Second, in studies that enrolled patients with iCCA+eCCA [14, 22, 23], the researchers did not report PD-L1 expression for iCCA and eCCA separately. Therefore, the data were extracted from those studies by including the patients with iCCA and eCCA as a whole group. Third, only one study with patients with GBC [17] was included in the analysis. Although we searched the literature by using keywords containing gallbladder cancer, only one study of GBC was included finally. Therefore, the prognostic impact of PD-L1 in patients with GBC could not be sufficiently explored. Fourth, all included studies were retrospective. Although we did not limit the eligible studies to be retrospective or prospective; the included studies were retrospective after literature selection. More prospective studies on this issue are still needed in the future.

In summary, our meta-analysis demonstrated that PD-L1 overexpression was associated with worse OS but not DFS of patients with BTC. The prognostic value of PD-L1 expression was significant for OS of patients with iCCA and eCCA. However, no significant correlation was observed between PD-L1 expression and clinical features of patients with BTC. These results indicate that PD-L1 could play a pivotal role as an effective factor of poor prognosis in patients with BTC. Nevertheless, as the study had several limitations, further large-scale, well-designed studies are needed to confirm our results.

## MATERIALS AND METHODS

### Search strategy

This meta-analysis was performed on the basis of the Preferred Reporting Items for Systematic Review and Meta-Analysis (PRISMA) guidelines [37]. The databases of PubMed, Embase, and Web of Science were thoroughly searched until October 2019 by using the following retrieval keywords: (“PD-L1” OR “B7-H1” OR “CD274” OR “programmed cell death ligand 1”) AND (“cholangiocarcinoma” OR “biliary tract cancer” OR “gallbladder cancer” OR “bile duct cancer” OR “hilar cholangiocarcinoma” OR “distal cholangiocarcinoma” OR “intrahepatic cholangiocarcinoma” OR “extrahepatic cholangiocarcinoma”) AND (“survival” OR “prognostic” OR “prognosis” OR “outcome”). Moreover, the reference lists were manually screened to collect potentially relevant studies. Ethical approval was not needed for this meta-analysis because it does not include individual patient information.

### Inclusion and exclusion criteria

The inclusion criteria for eligible studies were as follows: (1) BTC was diagnosed on histopathological examination; (2) immunohistochemistry (IHC) analysis of PD-L1 expression was conducted; (3) association between PD-L1 expression and OS and/or disease-free survival (DFS) was presented, or sufficient information was provided to compute the hazard ratio (HR) and 95% confidence interval (CI) [38]; (4) the PD-L1 expression in tumor cells was determined; and (5) the report was in the English language. The exclusion criteria for this meta-analysis were as follows: (1) duplicate studies; (2) reviews, case reports, letters, and meeting abstracts; (3) studies with insufficient data; and (4) non-English reports.

### Date extraction and quality assessment

Data from candidate studies were evaluated and extracted by two independent investigators (C.L. and X.P.). Any disagreements were resolved through discussion. The following information was extracted from eligible studies: the first author’s name, publication year, number of cases, country, ethnicity, study period, patient age, tumor type, detection method, cut-off value for high expression of PD-L1, treatment method, HR and the corresponding 95% CI for OS and DFS, and clinicopathological characteristics. The quality of the included studies was assessed by using the Newcastle-Ottawa Scale (NOS) [39]. The NOS included the 3 following main categories: selection (0–4 points), comparability (0–2 points), and outcome assessment (0–3 points). The NOS scores ranged from 0 to 9. Studies with NOS scores ≥6 were indicated to be of high quality.

### Statistical analysis

All statistical analyses were performed using Stata version 12.0 (Stata Corp LP, TX, USA). The HRs and 95% CIs were used to assess the prognostic role of PD-L1 expression on the survival of patients with BTC. An HR >1 with a p-value <0.05 indicated a poor prognosis in patients with PD-L1 overexpression. The odd ratios (ORs) with 95% CIs were calculated to determine the correlations between PD-L1 expression and clinicopathological parameters. Statistical heterogeneity among studies was determined using the Cochran Q-test and I-squared test [40, 41]. *I*^2^ values > 50% and p-values <0.10 were considered to indicate significant heterogeneity, and the random effects model was applied. Otherwise, a fixed-effect model was implemented. Subgroup analysis—stratified by tumor type, ethnicity, and sample size—was performed. Sensitivity analysis was conducted to assess the reliability of the results. Meta-aggression analysis was conducted to identify the source of heterogeneity. Publication bias was measured by using both the Begg’s test and Egger’s test. P-values <0.05 were considered statistically significant.

## Supplementary Material

Supplementary Figure 1
