# Supplementary material for: Prognostic role of programmed death-ligand 1 expression in patients with biliary tract cancer: a meta-analysis
Source: Aging (Albany NY). 2019 Dec 27;11(24):12568–80. doi: 10.18632/aging.102588 (PMC6949100; doi:10.18632/aging.102588)
Supplement: Supplementary Figure 1 [file aging-11-102588-s001..pdf]

SUPPLEMENTARY FIGURE

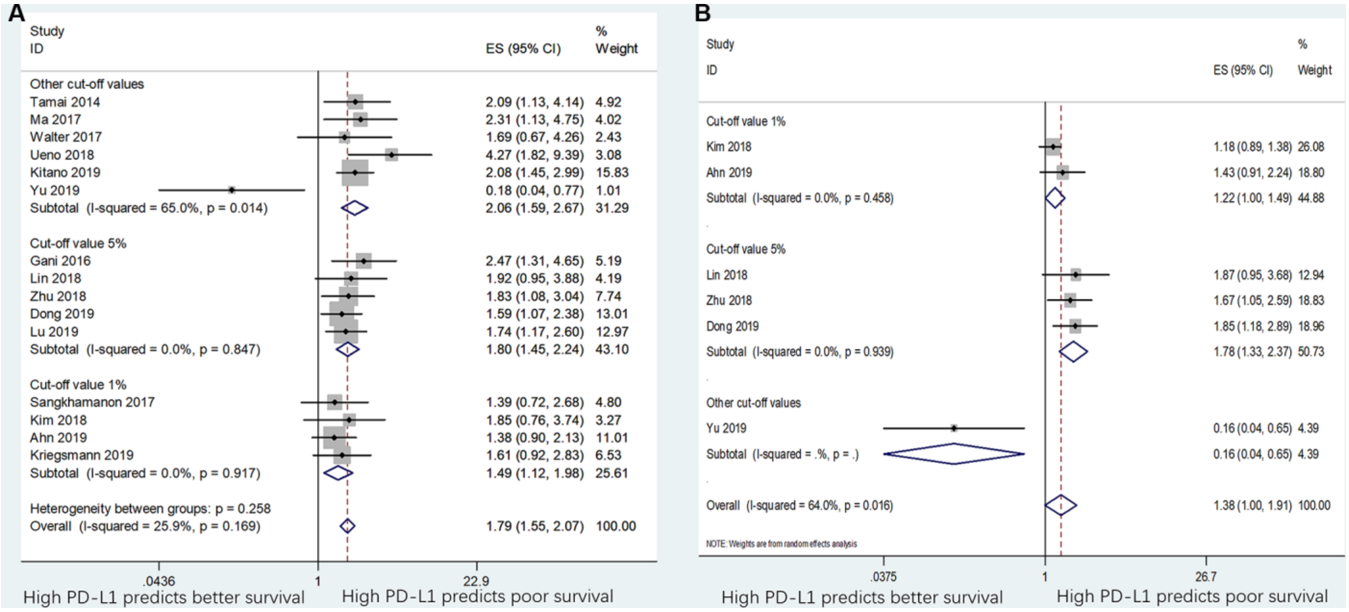

Supplementary Figure 1. Forest plots for the association between PD-L1 expression and (**A**) OS and (**B**) DFS categorized by different cut-off values: cut-off value 1%, cut-off value 5%, and other cut-off values. Note: The right-side means "High PD-L1 predicts poor survival" and the left-side means "High PD-L1 predicts better survival".
